# Cell cycle-dependent regulation of the bi-directional overlapping promoter of human BRCA2/ZAR2 genes in breast cancer cells

**DOI:** 10.1186/1476-4598-9-50

**Published:** 2010-03-04

**Authors:** Smita Misra, Shvetank Sharma, Anupriya Agarwal, Sheetal V Khedkar, Manish K Tripathi, Mukul K Mittal, Gautam Chaudhuri

**Affiliations:** 1 Division of Biomedical Sciences, Meharry Medical College, Nashville, TN 37208, USA

## Abstract

**Background:**

BRCA2 gene expression is tightly regulated during the cell cycle in human breast cells. The expression of BRCA2 gene is silenced at the G0/G1 phase of cell growth and is de-silenced at the S/G2 phase. While studying the activity of BRCA2 gene promoter in breast cancer cells, we discovered that this promoter has bi-directional activity and the product of the reverse activity (a ZAR1-like protein, we named ZAR2) silences the forward promoter at the G0/G1 phase of the cell. Standard techniques like cell synchronization by serum starvation, flow cytometry, N-terminal or C-terminal FLAG epitope-tagged protein expression, immunofluorescence confocal microscopy, dual luciferase assay for promoter evaluation, and chromatin immunoprecipitation assay were employed during this study.

**Results:**

Human *BRCA2 *gene promoter is active in both the forward and the reverse orientations. This promoter is 8-20 fold more active in the reverse orientation than in the forward orientation when the cells are in the non-dividing stage (G0/G1). When the cells are in the dividing state (S/G_2_), the forward activity of the promoter is 5-8 folds higher than the reverse activity. The reverse activity transcribes the ZAR2 mRNA with 966 nt coding sequence which codes for a 321 amino acid protein. ZAR2 has two C4 type zinc fingers at the carboxyl terminus. In the G0/G1 growth phase ZAR2 is predominantly located inside the nucleus of the breast cells, binds to the BRCA2 promoter and inhibits the expression of BRCA2. In the dividing cells, ZAR2 is trapped in the cytoplasm.

**Conclusions:**

*BRCA2 *gene promoter has bi-directional activity, expressing BRCA2 and a novel C4-type zinc finger containing transcription factor ZAR2. Subcellular location of ZAR2 and its expression from the reverse promoter of the BRCA2 gene are stringently regulated in a cell cycle dependent manner. ZAR2 binds to BRCA2/ZAR2 bi-directional promoter *in vivo *and is responsible, at least in part, for the silencing of BRCA2 gene expression in the G0/G1 phase in human breast cells.

## Background

The tumor suppressor protein BRCA2 is implicated in the regulated growth and proliferation of human breast [1-4], prostate [5,6], ovarian [7,8], esophageal [9], and pancreatic [10,11] cells. About 25% of autosomal dominant familial breast cancers are proposed to be caused by germline mutations in *BRCA2 *gene [12,13]. The mutations of *BRCA2 *gene predispose the cells towards neoplastic development. BRCA2 protein is over-expressed in most of the sporadic breast cancer cells [1-4]. The consequence of this over-expression of BRCA2 is not clearly understood. The notion could be that unique cellular mechanisms are triggered in the breast cancer cells to stimulate *BRCA2 *gene expression as a temporary measure to regulate the growth of the breast cancer cells. One potential mechanism of BRCA2 involvement in breast cancer progression may be through deregulation of the *BRCA2 *gene expression.

In humans, *BRCA2 *is a 3418-amino acid protein localized in the nucleus [14,15]. Loss of BRCA2 function has been shown to lead to centrosome amplification, chromosomal rearrangement, aneuploidy, and reduced efficiency of homologous recombination-mediated double-strand break repair. BRCA2 is known to directly bind to RAD51, BCCIP, PALB2, and BRAF35 proteins that are involved in meiotic/mitotic recombination, DNA double-strand break (DSB) repair, and chromosome segregation [1-4,15].

*BRCA2 *gene expression is stringently regulated during the cell cycle. BRCA2 expression is proportional to the rate of cell proliferation [14,16]. While BRCA2 expression is involved in cell cycle checkpoints and DNA repair, the mechanisms of cell cycle-dependent regulation of BRCA2 gene expression remains elusive. Analysis of the minimal promoter sequence of *BRCA2 *recognized several conserved binding sites for transcription factors such as E-box, E2F, and Ets recognition motifs [15,17-20]. USF1 and USF2 bind to the E-box [18,19] and Elf1, an Ets family protein, binds to the Ets recognition motifs [18] to activate *BRCA2 *gene expression [18,19]. Another transcription factor, NF-κB, has also been shown to bind to the promoter and induce *BRCA2 *gene expression [19]. The tumor suppressor protein TP53 represses the *BRCA2 *promoter by blocking the binding of USF [20]. Recently, poly-(ADP-ribose) polymerase-1 was reported to negatively regulate *BRCA2 *gene promoter by binding to it [15].

We have reported previously that the transcriptional repressor protein SLUG negatively regulates *BRCA2 *gene expression in SLUG-positive breast cancer cells by binding to an E2-box flanked by two Alu sequences in the -701 to -921-bp region [17,21]. Deletion of this sequence resulted in a 5-7-fold activation of the *BRCA2 *promoter. But the mechanism of cell cycle dependent regulation of BRCA2 gene expression in SLUG-negative cells remains unclear.

Here, we provide experimental evidence for the bi-directional activity of human *BRCA2 *gene promoter. We have shown here that the reverse activity of this promoter indeed transcribes a protein (ZAR1-like, we named ZAR2) that has significant similarity (36% identity), particularly at the C-terminal amino acid sequence of the C4-type zinc finger containing homeodomain protein, zygote arrest 1 (ZAR1) [22,23]. The similarity between ZAR1 and ZAR2 may indicate that these proteins belong to a unique family of transcriptional regulators. The chromosomal context of *BRCA2 *and *ZAR2 *genes are highly conserved among vertebrates studied. *BRCA2 *and *ZAR2 *gene expressions are reciprocally related during the cell cycle in human breast cells. Our studies suggest that negative regulation of BRCA2 gene expression by the ZAR2 at the G0/G1 phase of human breast cell growth may provide an additional mechanism of cell cycle-dependent regulation of its expression in both SLUG-positive and SLUG-negative cells.

## Methods

### Cell culture and synchronization

Human breast cancer cells were obtained from ATCC (Manassas, VA) and cultured in ATCC-recommended media [21,24]. We synchronized the cells by serum starvation and evaluated by FACS analysis, as described earlier [21]. For transfection and synchronization experiments, we transfected the cells with the plasmids (see below) and let them recover for 2 h in RPMI medium with 10% fetal bovine serum (FBS). This complete medium was then replaced with starvation medium (RPMI 1640, phenol red free, 0% FBS). After 36 h, the cells were stimulated to re-enter the proliferative cell cycle by replacing the starvation medium with medium containing 20% FBS. Cells were harvested at specific time points following serum stimulation and were processed. The progression of cells through the cell cycle during these experiments was monitored by flow cytometric analysis of replicate samples of propidium iodide-stained cells [18,21]. Each transfection experiment was repeated at least twice with triplicate samples each time. We used different human cell types including human mammary epithelial cells (HMEC), human breast cancer cells like MCF7, MDA-MB-468, MDA-MB-231, BT549, as described [21], for further verification and confirmation experiments.

### Promoter constructs, transfection and dual luciferase assay

**Figure 1:**
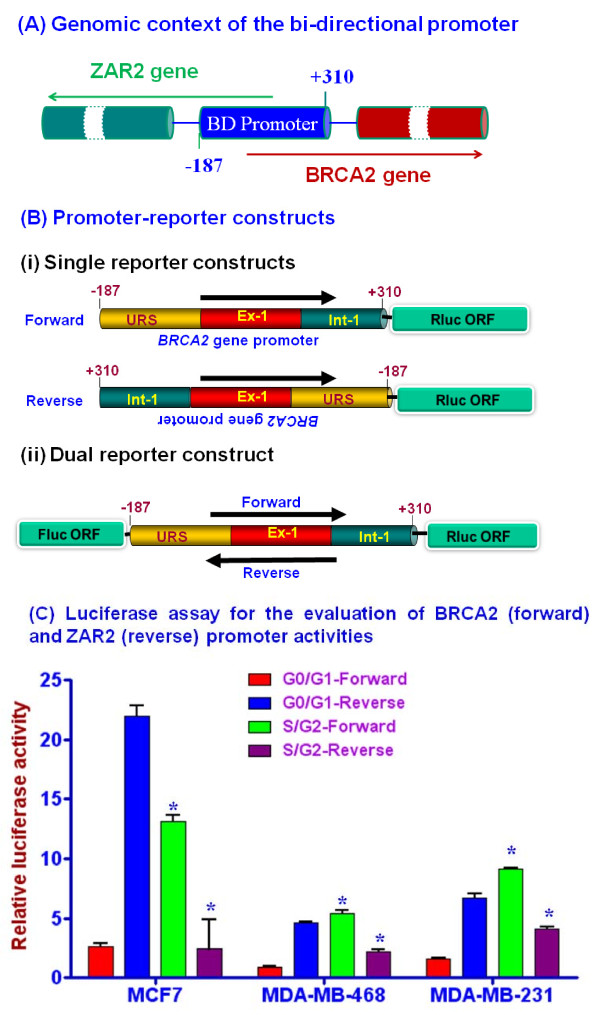
**Cell cycle dependent bi-directional activities of human BRCA2 gene promoter**. (A) Genomic context of human BRCA2 gene bi-directional (BD) promoter studied. The numbers shown are with respect to the transcription start site of human BRCA2 gene. (B) Maps of the reporter constructs in pRL-Null vector used in the study. (i) The single reporter constructs: pRL-FP (forward construct) and pRL-RP (reverse construct); (ii) the dual reporter construct. URS: upstream regulatory sequence; Ex-1: exon 1; Int-1: intron 1; Rluc: Renilla luciferase; Fluc: firefly luciferase; ORF: open reading frame. (C) Activities of the *BRCA2 *(forward) and the *ZAR2 *(reverse) promoters in different lines of human breast cancer cells at G0/G1 and S/G2 phases of their cell cycles. Results are mean ± SE (n = 6). The differences between the G0/G1 and S/G2 phase cells were statistically significant (shown by '*'; p < 0.001).

The human *BRCA2 *gene promoter (-187 to +310) was PCR amplified from the genomic DNA using primers P1 and P2 (Table 1), and cloned into pCR2.0-Topo (Invitrogen, Carlsbad, CA). The cloned insert (497 bp) was digested with Eco RI and cloned at the Eco RI site of the pRL-Null vector (Promega, Madison, WI). The resulting plasmids, pRL-FP (forward promoter) and pRL-RP (reverse promoter) (Fig. 1B), were transiently transfected into MCF7 cells using Lipofectamine 2000 transfection reagent (Invitrogen) along with the pGL3-control vector (Promega). Protein lysates were prepared from the cells, and dual-luciferase activities were measured as described previously [21,24]. *Renilla *luciferase (Rluc) activity was normalized with respect to firefly luciferase (Fluc) activity and presented as a ratio (relative light units; RLU). We also made a dual reporter construct (Fig. 1B) in which the BRCA2/ZAR2 promoter sequence is flanked by two reporter gene ORFs, Rluc is transcribed by forward (BRCA2) activity and Fluc is transcribed by the reverse (ZAR2) activity. To clone the firefly luciferase (Fluc) gene opposite to the Rluc gene in pRL-FP, Fluc ORF was taken out from pGL3-basic plasmid with Hind III and Sal I digestions and the DNA fragment was gel purified. The Fluc ORF was then cloned at the Hind III/Xho I sites of pRL-FP to obtain dual reporter plasmid pRL-DR. The identities of the plasmid constructs were verified by restriction mapping and nucleotide sequencing.

**Table 1:** Sequences of the oligonucleotides used in this study.

Oligo Name	Sequence (5'-3')	Purpose	Product size
P1	TCAGCGAGAAGAGAACAC	BRCA2/ZAR2 gene promoter amplification	497 bp
		
P2	TTGGCAGAGACAAAAGGGC		

P3	CGACTGGAGCACGAGGACACTGA	Gene racer ZAR2 5'-RACE	814 bp
		
P4	GCTGTTTGTGCCCTGAGAGTCC		

P5	AAGCTTATGGAGCGCTTTGTCCGT	PCR of C-terminal FLAG-tagged ZAR2	975 bp
		
P6	GGATCCCATCACATATTTAAAGCTGTAAA		

P7	AAGCTTATGGAGCGCTTTGTCCGT	PCR of N-terminal FLAG-tagged ZAR2	978 bp
		
P8	TCTAGATCACATCACATATTTAAAGCTGTAAAT		

P9	CACTGTAAAGATTGTAAGACCAGG	ZAR2 RT-PCR	140 bp
		
P10	CTGACATTGGATTGCTTCTACTCG		

P11	GTACAGGAAACAAGCTTCTGA	BRCA2 RT-PCR	271 bp
		
P12	GACTAACAGGTGGAGGTAAAG		

P13	GCTCGTCGTCGACAACGGCTC	Beta-actin RT-PCR	352 bp
		
P14	CAAACATGATCTGGGTCATCTTCTC		

P15	ACCCUUACAAGAGGGCGCAGCUUAA	siRNA#1	
		
P16	UUAAGCUGCGCCCUCUUGUAAGGGU		

**P17**	**UCCUGUGGCAAUAUUUACAGCUUUA**	siRNA#2	
		
**P18**	**UAAA GCUGUAAAUAUUGCCACAGGA**		

P19	ACCACAUGAGAGCGGCGACUUCUAA	Control siRNA#1	
		
P20	UUAGAAGUCGCCGCUCUCAUGUGGU		

**P21**	**UCCCGGUAUAAAUUUCGACUGUUUA**	Control siRNA#2	
		
**P22**	**UAAACAGUCGAAAUUUAUACCGGGA**		

**P23**	**ATCACTACTTGTCATCGTCATCCTTGTAGTCG**	FLAG reverse primer	

### GeneRacer analysis of ZAR2 gene transcription start site

The transcription start site of the *ZAR2 *gene was determined with the reagents from the GeneRacer Kit (Invitrogen). RNA was isolated from MCF7 cells using TRIzol reagent. The DNAse (RQ1, Promega)-treated RNA was then digested with calf intestinal phosphatase to remove 5'-phosphate from broken RNAs (if any). The 5'-caps of the intact mRNAs were removed by digestion with tobacco acid pyrophosphatase followed by ligation of a RNA oligonucleotide (44 nt) by T4 RNA ligase following suppliers protocols and using their reagents. These RNAs were used as template to make the cDNAs with GeneRacer oligo(dT) primer. The 5'-end of the ZAR2 mRNA was amplified using the GeneRacer 5'-primer and *ZAR2 *gene specific antisense primer (P3 and P4, Table 1) and Platinum Pfx DNA polymerase (Invitrogen). The PCR conditions were 1 cycle at 94°C for 2 min; 5 cycles at 94°C for 30 sec, 72°C for 2 min; 5 cycles at 94°C for 30 s, 70°C for 2 min; 25 cycles at 94°C for 30 sec, 60°C for 30 s, and 68°C for 2 min, and 1 cycle at 68°C for 10 min. The PCR product (814 bp: 770 bp from the ZAR2 mRNA + 44 bp from the RNA anchor) was gel purified, cloned and the nucleotide sequences were determined [21,24].

### Expression of 3X-FLAG tagged ZAR2 in MCF7 cells

Human *ZAR2 *(h*ZAR2*) gene ORF (NM_001136571; 1-966 bp) was PCR amplified from the cDNAs derived from MCF7 cells with Hind III and Bam HI site-containing primers (P5 and P6, Table 1). The reverse primer did not have the endogenous stop codon. The PCR product was cloned at the Hind III/Bam HI sites of p3xFLAG-CMV-14 vector (Sigma Chemical Co. St Louis, MO) to get the C-terminal FLAG tagged construct. For the expression of N-terminal FLAG-tagged ZAR2, ZAR2 ORF was amplified from the cDNAs derived from MCF7 cells with Hind III and Xba I site-containing primers (P7 and P8, Table 1). The reverse primer retained the endogenous stop codon. The PCR product was cloned at the Hind III/Xba I sites of p3xFLAG-CMV-10 vector (Sigma). The clones were sequence verified. The plasmid DNA was transfected in the MCF7 cells using Lipofectamine 2000 (Invitrogen) following supplier's protocol. After 36 h, cells were lyzed in TRIzol (Invitrogen) for RNA isolation or in Cell lytic reagent with protease inhibitors (Sigma) for Western blotting analysis [21,24]. Stable transfectants were also selected in G418 (1000 μg/ml) containing growth medium.

### Immunofluorescence analysis

Cells were transfected in 6-well plate with either the C-terminal FLAG-tagged construct (p3XFLAG-CMV14 or p3XFLAG-CMV14-ZAR2) or with the N-terminal FLAG tagged constructs (p3XFLAG-CMV10 or p3XFLAG-CMV10-ZAR2) using Lipofectamine 2000 (Invitrogen). After 16 h of transfection, cells were trypsinized and plated in 8-well chamber slides for 24 h in complete growth medium, washed with PBS, fixed with ice-cold methanol for 10 min and permeabilized in 50 mM NH_4_Cl and 0.2% Triton X100 in PBS. After blocking with 5% goat serum in PBS, the cells were incubated with anti-FLAG M2 monoclonal antibody (Sigma) in the blocking buffer overnight at 4°C [24]. After 16 h, the slides were washed 5 times with PBS and treated with secondary antibody conjugated with the red fluorescent dye (Alexa Fluor R555-conjugated donkey anti-mouse IgG, Invitrogen) for 1 h at room temperature. When performed dual labeling, unsynchronized MCF7 cells expressing C-terminal FLAG-tagged ZAR2 protein were processed similarly but incubated with both anti-FLAG M2 mouse monoclonal antibody (Sigma) and cyclin A monoclonal rabbit antibody (Abcam, Cambridge, MA) in the blocking buffer overnight at 4°C. After 16 h, the slides were washed 5 times with PBS and treated with secondary antibody conjugated with the red fluorescent dye (Alexa Fluor R555-conjugated donkey anti-mouse IgG, Invitrogen for FLAG) and the green fluorescent dye (Alexa Fluor R488-conjugated donkey anti-rabbit IgG, Invitrogen for cyclin A) for 1 h at room temperature. The cells were subsequently washed with PBS four times and stained with DAPI (Sigma) or Topro (Invitrogen). Finally, each slide was examined by confocal fluorescence microscopy (Nikon TE2000-U-CI confocal microscope). Each representative image was examined and digitally recorded at the same cellular level and magnification [24].

### Real time RT-PCR

Total RNA was isolated from cultured cells using TRIzol reagent (Invitrogen) according to the manufacturer's instructions. Isolated RNA was treated with DNase (RQ1, Promega) and then first strand cDNA was synthesized for real-Time RT-PCR (19). First strand cDNAs were synthesized using iScript cDNA synthesis kit (Biorad) with 5 μg of total RNA per reaction. For the real-time RT-PCR reaction cDNA (0.5 μl) was used per well in a total reaction volume of 25 μl. The iQSYBR green supermix (Biorad) containing the antibody-mediated hot start iTaq DNA polymerase was used for the PCR reaction. RT-PCR conditions were 1 cycle at 95°C for 3 min; 40 cycles at 95°C for 30 sec and 55°C for 1 min; 1 cycle at 95°C for 1 min; 1 cycle of 55°C for 1 min then 80 cycles for 10 sec each with 0.5°C increment after cycle two starting at 55°C, to collect the melt curve data and hold at 20°C [21,24]. The end point RT-PCR was done using Taq PCR master mix (Qiagen). PCR conditions were 1 cycle at 94°C for 5 min; 40 cycles: 94°C for 1 min, 55°C for 1 min and 72°C for 1 min; 1 cycle at 72°C for 10 min and then hold at 4°C.

### Quantitative Chromatin Immunoprecipitation (ChIP)-PCR Analyses (qChIP-PCR)

MCF7 cells stably transfected to over express C-terminal FLAG-tagged ZAR2 were used for this study. ChIP assays were done with the EZ MagnaChIP kit reagents and protocols (Upstate-Millipore). Briefly, cells were treated with formaldehyde (1% final concentration) for 10 min at 37°C. Cross-linking was terminated with addition of glycine (0.125 M final concentration). Cells were washed twice with ice-cold PBS containing protease inhibitor cocktail (Sigma). The chromatin pellets were sonicated in SDS lysis buffer [1% SDS, 10 mM EDTA, 50 mM Tris (pH 8.1)] to an average DNA size of 500 bp with a Fisher model 50 Sonic Dismembranator using an optimized sonication condition. The sonicated extract was centrifuged for 10 min at maximum speed and diluted with ChIP dilution buffer (0.01% SDS, 1.1% Triton X-100, 1.2 mM EDTA, 16.7 mM Tris-HCl (pH 8.1), 167 mM NaCl). The diluted ChIP lysates were pre-cleared with Magna beads for 30 min at 4°C. Immunoprecipitations were performed at 4°C overnight with either FLAG (Sigma) or normal mouse IgG (Santa Cruz Biotechnology). After 1 h incubation with 20 μl Protein A-Magna beads suspension, the conjugates were collected by magnetic separator. Immunocomplexes were washed twice sequentially in low salt immune complex wash buffer [0.1% SDS, 1% Triton X-100, 2 mM EDTA, 20 mM Tris-HCl (pH 8.1), 150 mM NaCl], high salt immune complex wash buffer [0.1% SDS, 1% Triton X-100, 2 mM EDTA, 20 mM Tris-HCl (pH 8.1), 500 mM NaCl], LiCl immune complex wash buffer [0.25 M LiCl, 1% IGEPAL-CA-630, 1% deoxycholic acid, 1 mM EDTA, 10 mM Tris (pH 8.1)], and TE buffer [10 mM Tris-HCl, 1 mM EDTA (pH 8.0)]. Elution of the immunocaptured chromatin complexes were performed with ChIP elution buffer provided in the kit. The DNA-protein cross-linking was reversed by incubating at 62°C for 2 h with Proteinase K. DNA fragments were obtained using Qiagen DNA purification column. DNA samples and standards were analyzed using real-time PCR system (BioRad) and iQSYBR Green PCR Master Mix (BioRad). Primers used to amplify BRCA2/ZAR2 promoter were as described [21] (Table 1). The following cycling parameters were used: 95°C for 3 min, 40 cycles of 95°C for 15 s, 55°C for 30 s, and 72°C for 1 min. Dissociation curve analyses were performed to confirm specificity of the amplification products. All samples were run in triplicate and all data were normalized with control IgG and 1% input DNA amplification [21,24].

### Knockdown of ZAR2 gene expression

ZAR2 mRNA was knocked down in MCF7 cells using 50 pmol/ml of ZAR2 specific stealth siRNA#1 (P15 and P16, Table 1) and stealth siRNA#2 (P17 and P18, Table 1). Corresponding control stealth siRNAs used were (#1: P19/P20 and #2: P21/P22, Table 1). Stealth siRNAs were custom designed and synthesized by Invitrogen. Transfection of the cells with siRNAs was done by lipofection following Invitrogen-provided protocol. After 68 h cells were lysed in TRIzol for RNA isolation. First strand cDNAs were synthesized using iSCRIPT cDNA synthesis kit (BioRad). Evaluation of the levels of ZAR2 and BRCA2 mRNAs was done by real-time RT-PCR using iQSYBR green Supermix (BioRad). To evaluate the effect of ZAR2 mRNA knockdown on the activities of the forward (BRCA2) and the reverse (ZAR2) promoters, MCF7 cells were transfected with 50 pmol/ml of the ZAR2 or control siRNA for 68 h. The cells were then transfected with the BRCA2 forward or reverse promoter containing reporter constructs (Fig. 1). Dual luciferase assay was performed after 24 h as described previously [21] following the supplier's protocol (Promega).

## Results

### Human BRCA2 gene promoter has bi-directional activity

We studied the minimal promoter of human *BRCA2 *gene as established by several groups [15,17-21] (Fig. 1A). We cloned the 497 bp promoter DNA sequence (-187 to +310) of human *BRCA2 *gene in front of *Renilla *luciferase (*Rluc*) gene in pRL-Null plasmid (Promega) to obtain clones with the insert either in the forward or in the reverse orientation with respect to the luciferase gene (Fig. 1B). We also developed a dual reporter construct in which the firefly luciferase (*Fluc*) gene ORF is transcribed by the reverse activity of the cloned promoter and the forward activity of the promoter will transcribe *Rluc *gene ORF (Fig 1B) from a single plasmid DNA. The single reporter construct with the forward orientation of the promoter yielded the *BRCA2 *gene promoter activity whereas the construct with the reverse orientation of the promoter revealed the activity of *ZAR2 *gene. We found that both the forward and the reverse orientations of the promoter have significant promoter activities (Table 2). Surprisingly, the activity of the reverse promoter was significantly higher in comparison to that of the forward promoter in different human breast cancer cells. These were unsynchronized cells with 90-95% confluency and the difference between the two activities varied depending upon the cell line tested (Table 2). When these cells were transiently transfected with the reporter constructs and the promoter activities were assayed at 40-60% confluency, the forward activity was higher than the reverse activity (data not shown), suggesting cell cycle dependency in the regulation of these promoters.

**Table 2:** Relative activities of the forward and the reverse promoters of BRCA2 gene in different unsynchronized human breast cancer cells at 95% confluency.

Promoter orientation	Relative luciferase activity* in			
	
	MCF-7	MDA-MB-468	MDA-MB-231	BT549
**Forward**	2.6 ±0.7	0.9 ± 0.2	1.6 ± 0.2	3.0 ± 0.5

**Reverse**	22.0 ± 2.1	5.4 ± 0.3	8.7 ± 0.8	15.4 ± 0.1

*Renilla luciferase activities are expressed as relative light units (RLU) normalized with respect to firefly luciferase activities from the same cellular extracts. Results are mean ± SE (n = 6).

### The forward and the reverse promoter activities are differentially regulated during the cell cycle

Regulation of BRCA2 gene expression during cell cycle progression is well-documented [14,18,19,21,25]. To evaluate whether the forward and the reverse promoter activities of BRCA2 gene promoter is regulated during the cell cycle, we synchronized different human breast cancer cell lines that were transiently transfected with either of the single-reporter plasmid constructs (Fig. 1B) and pGL3-Control plasmid (as a transfection control; Promega). Our data suggest that *ZAR2 *gene promoter activity is 2-7 folds higher than the *BRCA2 *gene promoter activity in the G0/G1 phase cells whereas it is significantly less than the *BRCA2 *gene promoter activity in the S/G2 phase cells (Fig. 1C). Similar results were obtained with MCF10A and HMEC cells. The ratio of the Rluc/Fluc from the cells transiently transfected with the dual-reporter construct (Fig. 1B) increased when the cells were shifted to S/G2 phase from G0/G1 phase (Table 3). These data are in agreement with the suggestion that these two promoters are reciprocally regulated during the cell cycle.

**Table 3:** Ratio of the forward (BRCA2) and the reverse (ZAR2) activity in the G0/G1 and S/G2 growth phases of different breast cancer cells using transient transfection with the dual reporter/promoter construct (see Fig. 1B).

Growth Phase	Ratio of Rluc/Fluc activities* in			
	
	MCF7	MDA-MB-468	MDA-MB-231	BT549
**G0/G1**	0.125 ± 0.03	0.23 ± 0.06	0.33 ± 0.01	0.28 ± 0.07

**S/G2**	3.25 ± 0.14	3.12 ± 0.11	2.5 ± 0.12	3.31 ± 0.13

*Results are mean ± SE (n = 6)

### The bi-directional promoter of BRCA2 gene produces overlapping transcripts

**Figure 2:**
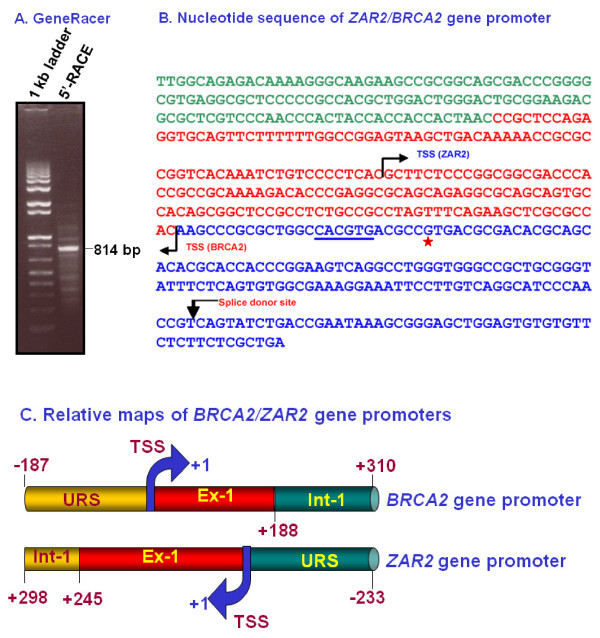
**The transcription start sites of the reverse transcript from BRCA2 gene bi-directional promoter**. (A) GeneRacer amplification product for ZAR2. See Materials and methods for details. (B) Nucleotide sequence of human ZAR2/BRCA2 bi-directional promoter. The transcriptional start sites (TSSs), as determined by GeneRacer technique, are shown. The segment in green color is the sequence complementary to part of the intron 1 sequence of human *BRCA2 *gene, the segment in red color is from exon 1 and the blue part is from the upstream sequence of *BRCA2 *gene. The E-box sequence essential for BRCA2 gene expression [18,19] is underlined. The splice donor site at the ZAR2 gene exon 1/intron 1 junction is indicated by a downward arrow. The 'G' residue at the SNP site at -26 from BRCA2 gene transcription start site is shown by a red *. (C) Cartoon showing the human *BRCA2 (upper panel) and ZAR2 *(lower panel) gene promoter studied. The identities of *ZAR2 *exon1 (Ex-1) and intron 1 (Int-1) were experimentally determined in this study. TSS: transcription start site (designated as +1); URS: upstream regulatory sequence.

Human *ZAR2 *gene is designated in the NCBI database as ZAR1L (#LOC646799). This gene is recently reported by *in silico *analysis in human as well as in bovine cells [22,23]. It is reported to have four exons; exon 1 starts with the translation start codon. The 5'-UTR sequence for ZAR2 mRNA has not yet been reported. Since we hypothesized that *ZAR2 *gene is transcribed from the *BRCA2 *gene promoter, the transcription start site of *ZAR2 *gene should also be located within the 497 bp promoter. To determine the 5'-UTR as well as the transcription start site of the *ZAR2 *gene, we employed GeneRacer technology (Invitrogen). We amplified the 5'-RACE product (814 bp: 770 bp from mRNA + 44 bp from the Gene Racer RNA anchor) (Fig. 2A) and determined the nucleotide sequence of the cloned insert. Alignment of the nucleotide sequence of the 5'-RACE product with genomic DNA sequence revealed that the transcription start site (TSS) of *ZAR2 *is located within the exon 1 of *BRCA2 *gene while the exon 1-intron 1 junction of *ZAR2 *is located within the upstream sequence of the *BRCA2 *gene (Fig. 2B). As a control for the GeneRacer technique, we verified the transcription start site and the first splice donor site of human *BRCA2 *gene in parallel. The locations of the transcription start sites and the first splice donor site at the reverse strand of the *BRCA2 *gene promoter are shown in Fig. 2B. Relative maps of the BRCA2 and ZAR2 transcription start sites (TSS) in the bi-directional promoter tested are shown in Fig. 2C. These data suggest that BRCA2 and ZAR2 transcripts have 111 nt complementary and antiparallel overlaps with each other at their 5'-ends (see below).

### The genetic arrangements of BRCA2 and ZAR2 genes are highly conserved among the vertebrates studied

**Figure 3:**
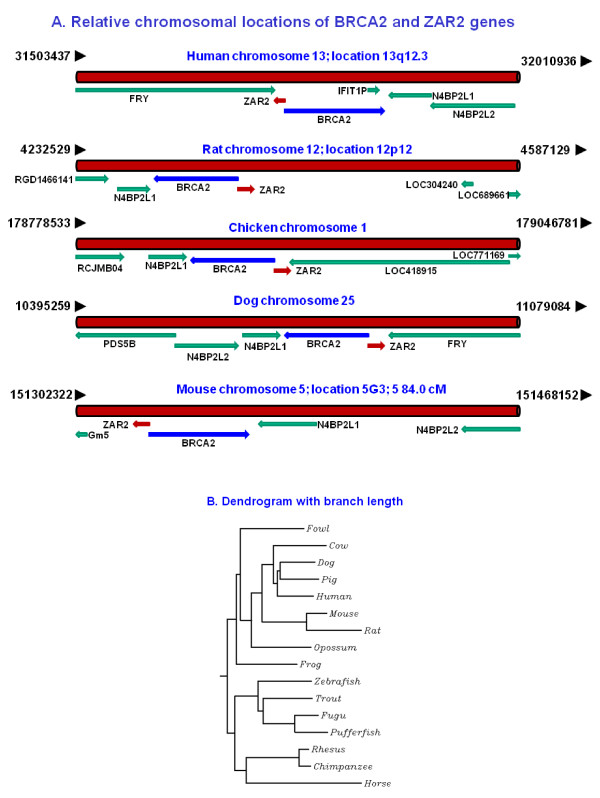
**Conservation of the BRCA2/ZAR2 genetic arrangements in vertebrates**. (A) Relative chromosomal locations of BRCA2 and ZAR2 genes in different vertebrates. The maps were obtained from NCBI site for Entrez genes http://www.ncbi.nlm.nih.gov/sites/entrez?db=gene&term=BRCA2+ Not drawn to the scale. (B) Dendrogram with branch lengths for the ZAR2 proteins from different vertebrates. The putative ZAR2 protein amino acid sequences were mined from the NCBI Entrez database and dendrogram with branch length was analyzed by CLUSTALW program http://align.genome.jp/.

The reciprocal relationship between BRCA2 and ZAR2 genes should be of significant biological importance because the bi-directional arrangements of these genes are highly conserved in several vertebrate species (Fig. 3A). Database analysis (NCBI GenBank) showed that although several other surrounding genes have changed in their relative positional arrangements, BRCA2/ZAR2 pair remains intact from bird to human and perhaps in others (Fig. 3A). Like BRCA2, ZAR2 is present in many vertebrates (Fig. 3B). Unlike BRCA2, which is found in many organisms from protozoa to human [26-29], we could not find any significant ortholog or paralog of ZAR2 mRNA or protein in non-vertebrates or microbes by database search.

### A new exon and a new intron are identified for human ZAR2 gene

**Figure 4:**
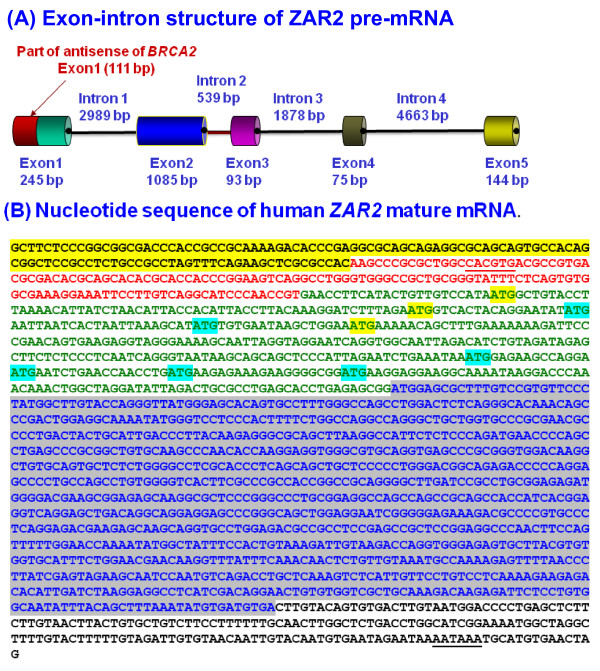
**The exon-intron structure and mRNA sequence of human *ZAR2 *gene**. (A) Cartoon showing the exon-intron structure of human *ZAR2 *gene. The first exon of *ZAR2 *overlaps with the exon 1 of the *BRCA2 *gene (not drawn to the scale). (B) Nucleotide sequence of human *ZAR2 *mature mRNA. The 5'-UTR sequence was experimentally determined (see text for details). The putative protein coding sequence (ORF) is shown in blue and highlighted in gray. The upstream AUG (uAUG) codons at the 5'-UTR are highlighted: out-of-frame uAUGs are in yellow shades; in-frame uAUGs are in green shades. The 5'-UTR sequence overlapping with *BRCA2 *mRNA sequences are shaded yellow. Rest of the 5'-UTR sequence of *ZAR2 *mRNA is derived from the newly identified exon 1 and is shown in red.

GeneRacer technology revealed a new exon and a new intron at the 5'-flank of the *ZAR2 *gene (Fig. 4A). The new exon 1 of *ZAR2 *is 245 bp, of which 111 bp and 134 bp are complementary and antiparallel to *BRCA2 *exon 1 and upstream sequences, respectively. With respect to *BRCA2 *gene transcription start site (TSS), the location of *ZAR2 *exon 1 is -134 to +111 (Fig. 2). Our study also revealed 431 bp additional sequences added before the translation start site of *ZAR2 *gene (Fig. 4B). Based upon these analyses, *ZAR2 *now has 5 exons and 4 introns (Fig. 4A) instead of 4 exons and 3 introns, as entered in the gene database. Our study revealed that the translation start site (ATG codon) is located within exon 2, instead of exon 1.

### The 5'-UTR of human ZAR2 mRNA is riddled with upstream AUG codons

The nucleotide sequence of the *ZAR2 *cDNA is shown in Fig. 4B. The 676 nt 5'-untranslated region (UTR) of the *ZAR2 *mRNA has several upstream AUG codons (uAUGs) and out of frame upstream open reading frames (uORFs). Thus, translation of *ZAR2 *mRNA may potentially be regulated by these uAUGs and uORFs [30,31].

### ZAR2 protein has strong similarity in amino acid sequence with ZAR1 and is highly conserved among vertebrates

**Figure 5:**
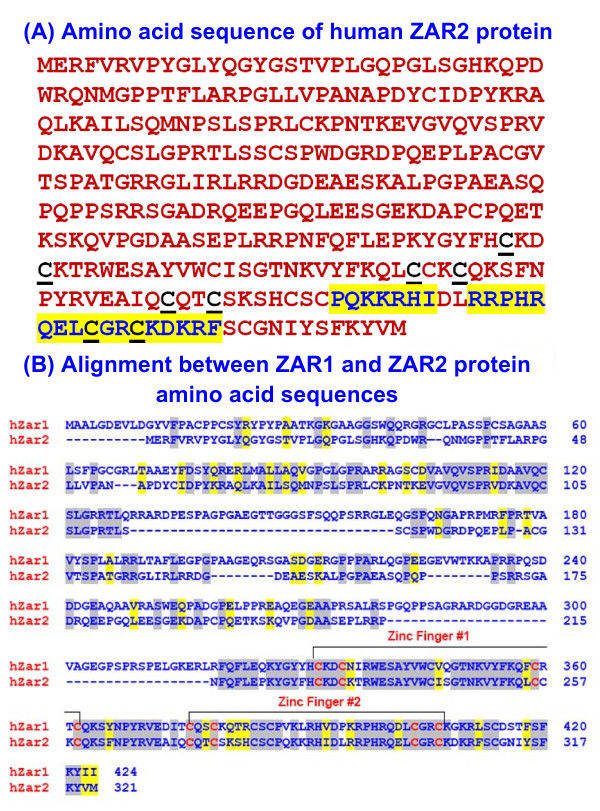
**Analysis of ZAR2 protein sequence**. (A) Amino acid sequence of human ZAR2 protein showing the C4 type zinc fingers. Cys residues of the zinc fingers are underscored and putative nuclear localization signals are highlighted. (B) CLUSTALW alignment between human ZAR1 (NP_783318) and ZAR2 (NP_001130043) amino acid sequences. hZar1: human ZAR1; hZar2: human ZAR2. Identical amino acid residues are highlighted in grey and the similar amino acid residues are shown in yellow shades. The conserved C4-type zinc fingers #1 and #2 are also shown.

**Figure 6:**
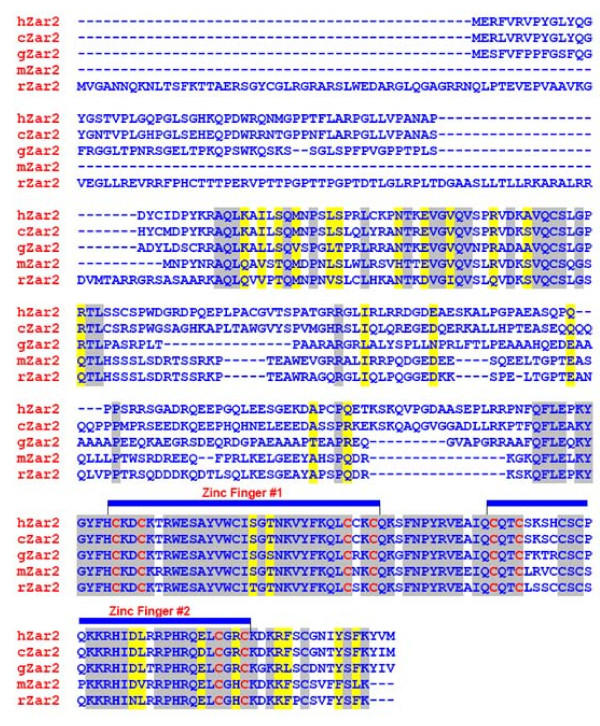
**CLUSTALW alignment of ZAR2 amino acid sequences from different vertebrates**. hZar2: human (*Homo sapiens*) ZAR2 (NP_001130043); cZar2: canine (*Canis familiaris*) ZAR2 (XP_534509); gZar2: chicken (*Gallus gallus*) ZAR2 (XP_001233594); mZar2: mouse (*Mus musculus*) ZAR2 (NP_001153165); rZar2: Rat (*Rattus norvegicus*) ZAR2 (XP_001071298). Identical amino acid residues are highlighted in grey and the similar amino acid residues are shown in yellow shades. The conserved C4-type zinc fingers #1 and #2 are also shown.

The 966 nt coding sequence of the *ZAR2 *mRNA codes for a 321 amino acid protein (Fig. 5A). We found two unique C4 type zinc fingers at the carboxyl terminus of this protein (Fig. 5A). These fingers are Cys-X2-Cys-X23-Cys-X2-Cys and Cys-X2-Cys-X24-Cys-X2-Cys where X is any amino acid. Such zinc fingers are found in nuclear receptors and GATA family of transcription factors among others [32]. The uniqueness of the ZAR2 zinc fingers is that they are comparatively longer (23-24 amino acids) than those of other proteins. The ZAR2 protein is largely similar with the ZAR1 protein, particularly at the C-terminus and the zinc finger Cys residues are highly conserved (Fig. 5B). The zinc fingers are also highly conserved among the ZAR2 orthologs from different animals (Fig. 6). These analyses suggest that ZAR2 is a potential transcription factor, perhaps binding to the promoters of its target genes through its C-terminal zinc finger domains. Alternatively, ZAR2 could be a non-DNA binding transcriptional regulator like FOG-1 [33] and perhaps regulates the function of *BRCA2 *gene promoter by restraining some regulatory protein.

### ZAR2 protein is predominantly located in the cytosol of unsynchronized human breast cells

**Figure 7:**
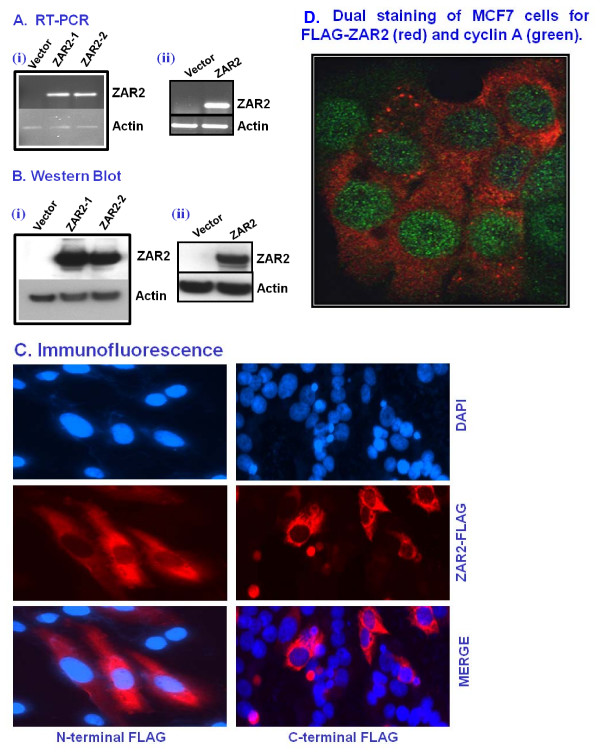
**Over expression of ZAR2 protein and its subcellular location in MCF7 and MDA-MB-231 cells**. (A) RT-PCR analysis showing the expression of N-terminal (i) and C-terminal (ii) FLAG-tagged *ZAR2 *mRNA in MCF7 cells. ZAR2-1 and ZAR2-2 are two independently derived transfectants. Similar results were obtained with MDA-MB-231 cells. (B) Western blotting analysis with anti-FLAG antibody showing expression of N-terminal (i) and C-terminal (ii) FLAG-tagged ZAR2 protein in MCF7 cells. Similar results were obtained with MDA-MB-231 cells. (C) Immunofluorescence analysis showing predominantly cytosolic location of N-terminal FLAG-tagged ZAR2 protein in the dividing MDA-MB-231 cells (left panel); and the C-terminal FLAG-tagged ZAR2 in dividing MCF7 cells (right panel). Anti-FLAG M2 antibody was used for the detection of FLAG-tagged ZAR2 protein in the cells. The cells were transiently transfected with the expression plasmid constructs and thus not all cells are expressing the recombinant protein. (D) Immunofluorescence confocal microscopy after dual labeling of the unsynchronized C-terminal FLAG-tagged ZAR2-expressing MCF7 cells with reagents for FLAG-ZAR2 (red) and the S-phase marker cyclin A (green). Predominant levels of FLAG-ZAR2 in the cytosol of the cells that have high levels of cyclin A in the nucleus. Nucleus was stained with Topro for confocal microscopy.

Subcellular location of a protein often reflects upon its biological function. To understand subcellular location of ZAR2 in human breast cells, we expressed N-terminal and C-terminal FLAG-tagged ZAR2 in these cells (Fig. 7A and 7B). Expression of both of these tagged proteins was necessary because we did not know whether tagging will compromise the subcellular localization of this protein. This was particularly important since the putative nuclear localization signals of ZAR2 are located at the C-terminal region of this protein (Fig. 5A). RT-PCR analysis with *ZAR2 *specific primer (P9, Table 1) and FLAG-tag specific primer (P23, Table 1) showed significant expression of the recombinant *ZAR2 *transcripts in the cells transiently transfected with either of the constructs (Fig. 7A). Similar data were obtained with Western blot analysis for the FLAG-tagged ZAR2 protein with anti-FLAG monoclonal antibody (Fig. 7B). Although ZAR2 is a zinc finger protein with attributes of a nuclear protein and has putative strong nuclear localization signals (Fig. 5A), our immunofluorescence microscopy analysis data with FLAG antibody showed predominant cytosolic localization of the ZAR2 protein in the transiently transfected human breast cells (Fig. 7C). This is true for both N-terminal as well as C-terminal FLAG-tagged ZAR2 proteins (Fig. 7C). Few cells in the population had significant levels of FLAG-tagged ZAR2 inside the nucleus (Fig. 7C). In an unsynchronized population of MCF7 cells transfected with FLAG-tagged ZAR2 construct also showed major presence of FLAG-ZAR2 (red) in the cytosol while the S-phase marker cyclin A (green) is predominantly located in the nucleus of the cell (Fig. 7D).

### Expressions of BRCA2 and ZAR2 during cell cycle are inversely related

**Figure 8:**
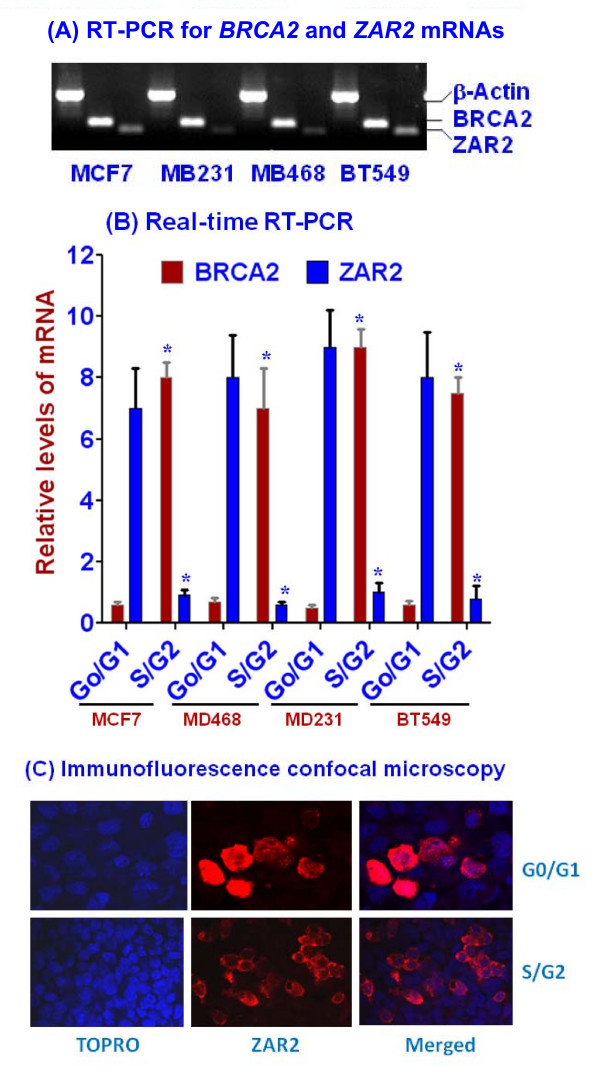
**Relative expressions of *BRCA2 *and *ZAR2 *mRNAs at different cell cycle stages of human breast cells**. (A) RT-PCR analysis showing the expressions of *BRCA2 *and *ZAR2 *mRNAs in the unsynchronized (mostly dividing) cells. β-Actin mRNA was used as a loading control. (B) Real-time RT-PCR evaluation of the relative levels of BRCA2 and ZAR2 mRNAs in different human breast cancer cells at G0/G1 and S/G2 phases. The differences between the G0/G1 and S/G2 phase cells were statistically significant (shown by '*'; p < 0.001). (C) Immunofluorescence confocal microscopy showing growth phase-dependent localization of N-terminal FLAG-tagged ZAR2 protein in the synchronized MCF7 cells. Anti-FLAG M2 antibody was used for the detection of FLAG-tagged ZAR2 protein in the cells. The cells were transiently transfected with the expression plasmid constructs and thus not all cells are expressing the recombinant protein.

RT-PCR analysis showed the expression of *BRCA2 *and *ZAR2 *mRNAs in unsynchronized dividing human breast cells (Fig. 8A). To evaluate whether the expressions of *BRCA2 *and *ZAR2 *genes at G0/G1 and S/G2 phases follow the pattern of their promoter activities, we measured the mRNA levels by real-time RT-PCR. In all the cells tested BRCA2 and ZAR2 expressions are inversely related. ZAR2 mRNA levels are higher at the G0/G1 phase with significant lower levels of BRCA2 mRNA (Fig. 8B). On the other hand, at the S/G2 phase the levels of BRCA2 mRNA are significantly higher than those of ZAR2 mRNAs (Fig. 8B).

### ZAR2 is predominantly located in the nucleus of G0/G1 phase human breast cells

We synchronized C-terminal FLAG-tagged ZAR2-expressing MCF7 cells and evaluated the subcellular location of this protein at G0/G1 and S/G2 phase by immunofluorescence confocal microscopy using FLAG antibody. Our data suggest that ZAR2 protein is predominantly concentrated in the nucleus of these cells at the G0/G1 phase whereas it is mainly present in the cytosol at the S/G2 phase cells (Fig. 8C). Evaluation of ZAR2 protein distribution in the subcellular fractions by Western blotting analysis also revealed similar localization pattern (data not shown). These data suggest that not only the expression of ZAR2 gene is strictly regulated cell cycle-dependently but its subcellular localization is also controlled in a growth stage-dependent manner. ZAR2 and BRCA2 gene expressions are thus inversely related.

### ZAR2 binds to the BRCA2/ZAR2 gene promoter in vivo

**Figure 9:**
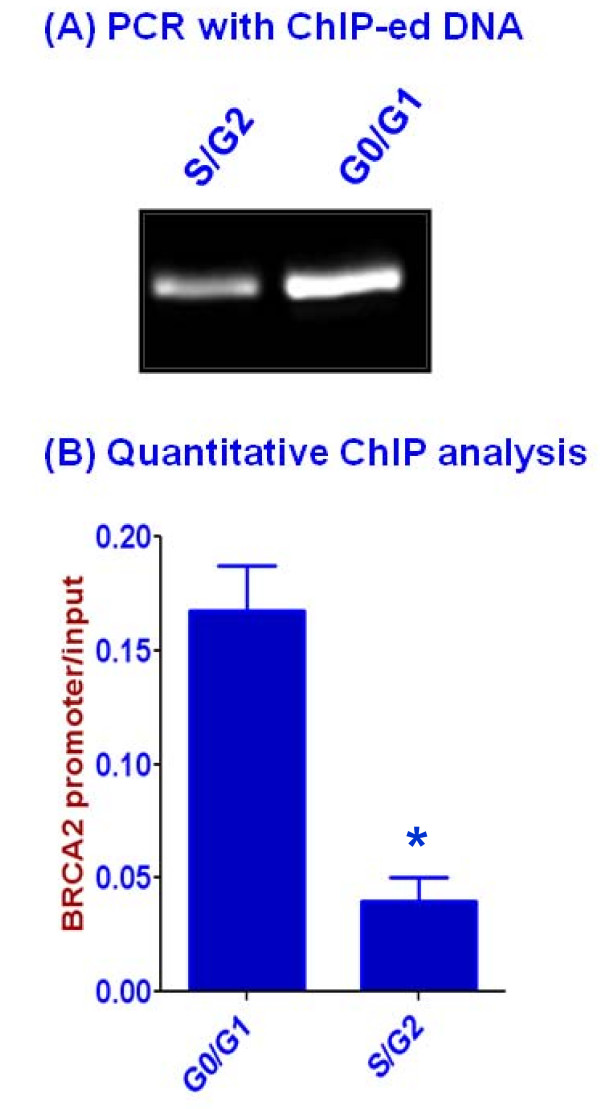
***In vivo *binding of ZAR2 protein to the BRCA2/ZAR2 gene promoter**. (A) PCR amplification of the immunoprecipitated chromatin DNA fragment pulled down with FLAG antibody from synchronized MCF7 cells over-expressing C-terminal FLAG-tagged ZAR2 protein at the G0/G1 and S/G2 phases. Input DNA (5% was used as control. Chromatin DNA fragments mock precipitated with mouse IgG did not significantly amplified any detectable DNA. BRCA2 gene promoter specific primers [21] were used for PCR amplifications. (B) Quantitative ChIP analysis of ZAR2 recruitment to BRCA2/ZAR2 bi-directional promoter in MCF7 cells at G0/G1 and S/G2 phases. qChIP-PCR analyses were performed with chromatin extracts harvested from cells over expressing C-terminal FLAG-tagged ZAR2. The mean values from triplicate data points are plotted and error bars indicate ± SE. The amplification values are normalized by subtraction with IgG control antibody and then division with 1% input DNA. Data shown were representative of three independent experiments (mean + SE) and the difference between the G0/G1 phase and the S/G2 phase cells was statistically significant (shown by '*'; p < 0.001).

**Figure 10:**
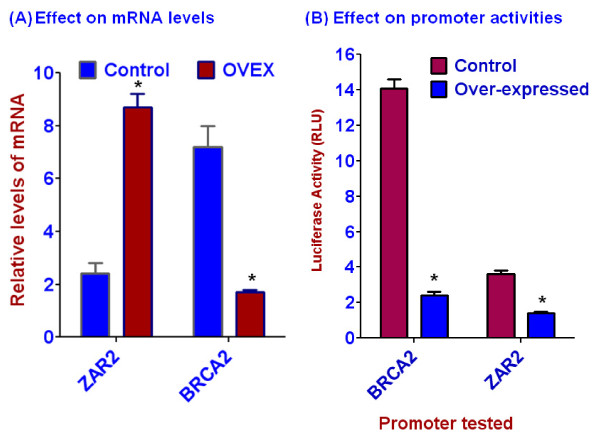
**Effects of over-expression (OVEX) of the C-terminal FLAG-tagged ZAR2 in synchronized MCF7 cells on the BRCA2 and ZAR2 mRNA levels (A) and on the activities of BRCA2 and ZAR2 gene promoters (B) at the S/G2 phase**. MCF7 cells were stably transfected with C-terminally FLAG-tagged ZAR2 and evaluated for their ZAR2 over expression. Levels of the mRNAs were determined by real-time RT-PCR [21]. Promoter activities were measured in MCF7 cells transiently transfected with the single-reporter constructs (Fig. 1B) following the dual luciferase assay protocols (Promega). pGL3-Control was used as normalization control as described in the 'Methods' section. Results are mean ± SE (n = 6). '*' indicates the difference between the corresponding control and the experimental sets is statistically significant (p < 0.001).

In order to understand the role of cell cycle-dependent regulation of ZAR2 gene expression on BRCA2 levels, we explored whether the C4-type zinc finger protein ZAR2 can bind to the BRCA2 promoter DNA. We used MCF7 cells over expressing C-terminal FLAG-tagged ZAR2 and employed quantitative ChIP techniques using commercially available FLAG antibody for this purpose. We found that ZAR2 binds tightly with BRCA2/ZAR2 bi-directional promoter preferentially at the G0/G1 phase of MCF7 cells (Fig. 9A and 9B). The nature (direct or indirect) and exact site(s) of binding of the ZAR2 protein to this promoter are yet to be determined. The outcome of over expression of ZAR2 and its binding to the BRCA2 promoter is not yet known. We found that over expression and DNA-binding of ZAR2 is associated with decrease in the level of BRCA2 mRNA (Fig. 10A) as well as inhibition of both BRCA2 and ZAR2 promoter activities (Fig. 10B), particularly at the S/G2 phase of MCF7 cells. Correlation between ZAR2 binding to the promoter and the repression of BRCA2 gene expression needs to be validated by rigorous mutational analyses.

### Knockdown of ZAR2 in the G0/G1 phase stimulated the expression of BRCA2

**Figure 11:**
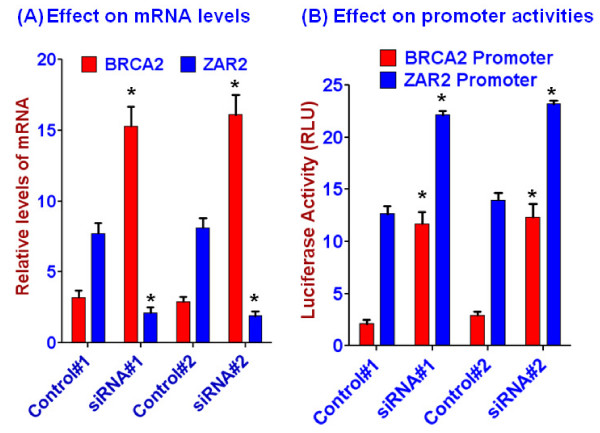
**Effect of knockdown of ZAR2 in synchronized MCF7 cells on the BRCA2 and ZAR2 mRNA levels (A) and on the activities of BRCA2 and ZAR2 gene promoters (B) at the G0/G1 phase**. ZAR2 was knocked down in MCF7 cells with two different double-stranded stealth siRNAs (Invitrogen). Levels of the mRNAs were determined by real-time RT-PCR. Promoter activities were measured in MCF7 cells transiently transfected with the single-reporter constructs (Fig. 1B) following the dual luciferase assay protocols (Promega). pGL3-Control was used as normalization control as described in the 'Methods' section. Results are mean ± SE (n = 6). '*' indicates the difference between the corresponding control and the experimental sets is statistically significant (p < 0.001).

To verify further the potential role of ZAR2 in the regulation of BRCA2 gene expression we knocked down the expression of ZAR2 by two different siRNAs designed from its ORF. When the mRNA levels of ZAR2 were knocked down to 50-55% at the G0/G1 phase of MCF7 cells, the levels of BRCA2 mRNA went up significantly (3.5-4 folds) (Fig. 11A). Interestingly, the activities of both of the ZAR2 and BRCA2 promoters were increased in the ZAR2 knocked down cells (Fig. 11B). Increased expressions of ZAR2 mRNA may explain why we could not down regulate the ZAR2 mRNA levels more than 50% with the siRNAs.

## Discussion

BRCA2 levels go up in many aggressively growing breast cancer cells [17-21]. It appears that the level of BRCA2 protein in the cell must commensurate with the need of the cells to avoid detrimental consequences in the cellular physiology. No BRCA2 in the dividing breast cells will predispose them to non-homologous end joining mode of DNA double strand break repair thus to potential oncogenesis [34]. Understanding the mechanisms of this stringent mechanism of *BRCA2 *gene expression regulation is critical to evaluate etiology of human breast cancer.

Human genome is riddled with bi-directional promoters [23,35,36]. In this study we characterized the bidirectional promoter that expresses *BRCA2 *and *ZAR2 *genes. Human BRCA1 gene and about 11% of the total other human gene promoters have bi-directional activities [36]. While assessing the activities of human *BRCA2 *gene promoter (Fig. 1A) in both orientations, reverse orientation serving as a negative control, we made three significant observations: (i) The human *BRCA2 *gene promoter is active in both the forward and the reverse orientations; (ii) The *BRCA2 *gene promoter is more active in the reverse orientation than in the forward orientation when the cells are in the non-dividing stage (G0/G1), and (iii) when the cells are in the dividing state (S/G_2_), the forward activity of the promoter is higher than the reverse activity (see below). The reverse activity was insignificant when we did not include the exon 1 and part of the intron 1 sequence of the *BRCA2 *gene (26). We have repeated this experiment with different human cell types including human mammary epithelial cells (HMEC), human breast cancer cells like MDA-MB-468, MDA-MB-231, BT549, immortalized human breast cells like MCF10A, MCF10AT, human liver cells HepG2 and human monocytes U937. In all these cells the promoter behaved similarly. Thus, we believe that this cell cycle dependent differential bi-directional promoter activity of the *BRCA2 *gene is an intrinsic property of *BRCA2 *and *ZAR2 *genes. Recently, a ZAR2 paralog, Xzar2, has been cloned from the African clawed frog *Xenopus laevis *[36]. Xzar2 was shown to be involved in epidermal fate determination mainly through signaling pathways distinct from that of BMP-Smad during early embryogenesis [37].

As mentioned above, *BRCA2 *gene expression is tightly regulated in human breast cells [14,15,17-21]. The *BRCA2 *mRNA and protein are only significantly expressed in the S/G2 phase cells and they are undetectable in the G0/G1 phase cells [18-21]. Over expression of BRCA2 protein was shown to be lethal for the survival of human pancreatic cancer cell line Capan-1 [38].

Several mechanisms are known to be operative in breast cancer cells to regulate BRCA2 gene expression [15,18-21]. We reported previously that cell cycle stage-dependent regulation of *BRCA2 *gene expression in SLUG-positive breast cells occurs through a distal E2-box/Alu repeat containing silencer element located upstream of the *BRCA2 *gene transcription start site [17,21]. The zinc-finger transcriptional repressor, SLUG, binds to the uniquely located E2-box sequence in the silencer element in the non-dividing cells and blocks the expression of *BRCA2 *gene by chromatin remodeling [21]. We recently found that peroxiredoxin 5 competes with SLUG for the binding to the *BRCA2 *gene silencer in the dividing cells and thus de-silences the expression of *BRCA2 *gene in the dividing human breast cells (Misra, S. and Chaudhuri, G., unpublished). Transcription factors other than SLUG that have been reported to regulate human *BRCA2 *gene expression include USF1 and 2 [18-20], P53 [20], NFkB [19], ElF1 [18], and PARP1 [15]. A recent report indicated the presence of a SNP (G to A) at the -26 position of human *BRCA2 *gene [39]. This SNP is in the exon 1 of ZAR2 gene (Fig. 2B). Whether TP53 also regulates *ZAR2 *gene expression and whether this SNP affects its promoter activity is yet to be determined.

The bi-directional promoter of BRCA2/ZAR2 gene produces two partially overlapping transcripts. Whether these RNAs hybridize with each other and form double-stranded (ds) RNA and whether this ds-RNA has any role in regulating the activities of the promoter is yet to be determined. One of the potential roles of the ds-RNA could be siRNA-mediated transcriptional gene silencing through DNA methylation [40,41].

The biological function of ZAR2 protein is not known. It has two putative C4-type zinc fingers and potentially could be a transcription factor. We found *BRCA2 *and *ZAR2 *gene expressions have inverse relationships during the cell cycle. It is possible that ZAR2 protein somehow inhibits *BRCA2 *gene expression. Although ZAR2 protein has two putative NLS sequences, in the dividing stage of the human breast cells ZAR2 is trapped predominantly in the cytoplasm. Thus, ZAR2 in the dividing breast cells may not have any significant effect on the *BRCA2 *gene expression. At the non-dividing (G0/G1) phase ZAR2 protein predominantly accumulates in the cell nucleus, binds to the BRCA2/ZAR2 gene promoter and consequently, both ZAR2 and *BRCA2 *gene expressions are inhibited. While it is tempting to speculate that ZAR2 represents a mechanism of cell cycle dependent regulation of BRCA2 gene expression, direct involvement of *ZAR2 *in *BRCA2 *gene transcription is yet to be determined.

As ZAR2 over expression decreased the levels of BRCA2 in the cells, this gene, if disregulated, and over expressed in the cells, it may promote the growth of the tumor. On the other hand, ZAR2 may be needed to suppress BRCA2 expression in the quiescent cells. Expression of BRCA2 in these cells could be detrimental for the cell growth and survival [37]. We made an interesting observation while knocking down ZAR2 mRNA levels in different breast cancer cells. Out of four cell lines tested (MCF7, MDA-MB-231, MDA-MB-468 and BT549), only BT549 died at the G0/G1 phase in the ZAR2 knocked down cells. We found that ZAR2 knockdown in the quiescent cells leads to the elevation of the levels of BRCA2 which should be detrimental to the cells [38]. But the ability to suppress the growth of the cells by BRCA2, the cell may need to have high MAGE-D1 level [42]. Our explanation for the essentiality of ZAR2 in the BT549 cells is that only these cells among the four cells tested have high levels of MAGE-D1 [42]. ZAR2 protein thus may have multiple balancing roles in the biology of BRCA2 and perhaps other molecules in the cells.

## Conclusions

*BRCA2 *gene promoter has bi-directional activity, expressing BRCA2 and a novel C4-type zinc finger-containing transcription factor ZAR2. BRCA2 and ZAR2 levels in the cells are inversely related with respect to the cell cycle. Subcellular location of ZAR2 and its expression from the reverse promoter of the BRCA2 gene are stringently regulated in a cell cycle dependent manner. ZAR2 accumulates in the nucleus of the cells at the quiescent stage of the cells and binds to the BRC2 gene promoter *in vivo*. ZAR2 is responsible, at least in part, for the silencing of BRCA2 gene expression in the G0/G1 phase in human breast cells.

## Abbreviations used

The abbreviations used are: ChIP: chromatin immuno precipitation; *ZAR1*: zygote arrest 1; *ZAR2*: ZAR1-like protein; UTR: untranslated region; RT-PCR: reverse transcriptase-PCR; uORF: upstream open reading frame.

## Competing interests

The authors declare that they have no competing interests.

## Authors' contributions

SM, SS, AA, SVK, MKT, and MKM participated in the acquisition of data. SM and GC were involved with the study concept and design. SM and GC contributed to the statistical analyses. SM, MKM, and GC participated in manuscript preparation. All authors participated in the interpretation of results and critical revision of the manuscript for important intellectual content. All authors read and approved the final manuscript.
